# Effect of device and expiratory maneuver technique on peak expiratory flow measurements

**DOI:** 10.1111/cpf.70067

**Published:** 2026-05-15

**Authors:** Leon L. Csonka, Antti Tikkakoski, Jussi Karjalainen, Lauri Lehtimäki

**Affiliations:** ^1^ Faculty of Medicine and Health Technology Tampere University Tampere Finland; ^2^ Department of Clinical Physiology and Nuclear Medicine Tampere University Hospital Tampere Finland; ^3^ Allergy Centre, Tampere University Hospital Tampere Finland

**Keywords:** asthma, device comparison, home monitoring, microspirometer, PEF meter, spirometer

## Abstract

**Background:**

Peak expiratory flow (PEF) obtained from dedicated PEF meters and various types of spirometers is often used interchangeably in research, despite differences in both devices and expiratory maneuver technique. The aim of this study was to assess the effects of expiratory maneuver type and measurement device on PEF.

**Methods:**

We recruited 20 healthy adults experienced in performing spirometry. Each subject performed three measurements using two expiratory maneuvers (a short, explosive maneuver and a long, spirometry‐style maneuver) with four devices: the handheld microspirometers Medikro Duo and MIR Spirobank, the laboratory spirometer Vyntus Pneumo, and the Mini‐Wright PEF meter.

**Results:**

PEF differed significantly between devices (F(3,133) = 171.8, *p* < 0.001), but not between short and long expiratory maneuver techniques (F(1,133) = 2.24, *p* = 0.137). There was no significant interaction between device and technique (F(3,133) = 0.72, *p* = 0.543). Compared with the Mini‐Wright, Spirobank yielded PEF values that were on average 87.1 L/min higher (74.6–99.7, *p* < 0.001), and Vyntus Pneumo 26.5 L/min higher (14.0–39.1, *p* < 0.001), whereas Medikro did not differ significantly from the Mini‐Wright (mean difference −9.5 L/min, −22.1–3.1, *p* = 0.269). The pairwise differences between the three spirometers were all statistically significant (all *p* < 0.001).

**Conclusions:**

Because short and long expiratory maneuver techniques yield similar results on PEF, future studies of home spirometry may compare the diagnostic performance of PEF and forced expiratory volume in 1 s (FEV_1_) using the same long, spirometry‐style expiratory maneuvers. However, comparison of absolute PEF values between devices must be done cautiously.

## 1 INTRODUCTION

Asthma is one of the most common chronic diseases worldwide and is characterized by variable expiratory airflow limitation (GINA, 2025; Vos et al., 2017). Objective assessment of lung function is central to its diagnosis and monitoring, most commonly using spirometry and peak expiratory flow (PEF) measurement. Spirometry measures several lung function parameters, including PEF, forced expiratory volume in 1 s (FEV_1_), forced expiratory volume in 6 s (FEV_6_), and forced vital capacity (FVC), whereas dedicated PEF meters record only PEF and are primarily used for home monitoring in primary care (GINA, 2025).

In addition to dedicated PEF meters, home PEF monitoring is increasingly performed using digitally recording handheld microspirometers. Although PEF derived from laboratory spirometry is less commonly used for clinical decision‐making because FEV_1_ is considered a more robust physiological parameter (Csonka et al., 2024, 2025), spirometric PEF values are frequently reported, compared, or pooled in research studies. However, devices differ in several respects, including sensor technology, sampling frequency, signal processing, flow resistance, and calibration, which may lead to systematic differences in measured PEF (Miller et al., 2005). Although several studies have already reported differences in measured PEF between dedicated PEF meters, possible differences in PEF derived from spirometers remain less well studied (Folgering et al., 1998; Koyama et al., 1998; Takara et al., 2010).

The expiratory maneuver used to obtain PEF also differs between measurement contexts. PEF meters are typically used with a short, explosive maneuver, whereas spirometry requires a maximal forced expiration sustained long enough to record FEV_6_ or FVC. Although some studies suggest that PEF obtained during a full spirometric maneuver may be slightly lower than PEF obtained using a brief, explosive maneuver, the available evidence remains limited, and the clinical relevance of any such difference is unclear (Agarwal & Gupta, 2007; Bongers & O′Driscoll, 2006; Wensley et al., 2000).

Because PEF values obtained using different devices and maneuver techniques are often used interchangeably, it is important to understand how these factors influence measured PEF. The aim of this study was to assess the effects of measurement device and expiratory maneuver technique on PEF in healthy subjects using a laboratory spirometer, two handheld microspirometers, and a dedicated PEF meter.

## 2 METHODS

We recruited 20 healthy volunteers from the staff of Tampere University Hospital to perform PEF measurements. The subjects were experienced in performing lung function measurements and had good blowing technique, which reduced within‐subject variability and allowed us to focus on differences between expiratory maneuver techniques and devices. Each subject performed three measurements using two expiratory maneuvers (a short, explosive PEF maneuver and a long, spirometry‐style maneuver) with four different devices: the handheld microspirometers Medikro Duo M920 (Medikro Oy, Kuopio, Finland) and MIR Spirobank II Smart (Medical International Research S.p.A., Rome, Italy), the laboratory spirometer Vyntus Pneumo (Jaeger Medical GmbH, Höchberg, Germany), and the Mini‐Wright PEF meter (Clement Clarke International Ltd., Mountain Ash, Wales, UK). All measurements were performed under supervision, and the technical acceptability and repeatability of measurements were assessed according to European Respiratory Society (ERS) criteria (Graham et al., 2019). For each subject, device, and maneuver combination, the mean of the three measurements was used for statistical analysis.

Data analysis was performed using IBM SPSS Statistics for Windows, Version 29 (IBM Corp., Armonk, NY, USA). Data are presented as estimated marginal means with 95% confidence intervals (CIs) unless otherwise stated. The effects of measurement device and expiratory maneuver technique on PEF were analyzed using a linear mixed‐effects model with device and technique and their interaction as fixed effects and subject as a random effect. Model parameters were estimated using restricted maximum likelihood. For the linear mixed‐effects model, unadjusted p‐values were used, and *p* < 0.05 was considered statistically significant. For post hoc pairwise comparisons, Bonferroni‐adjusted *p*‐values were used, with adjusted *p*‐values < 0.05 considered statistically significant.

## 3 RESULTS

**Figure 1:**
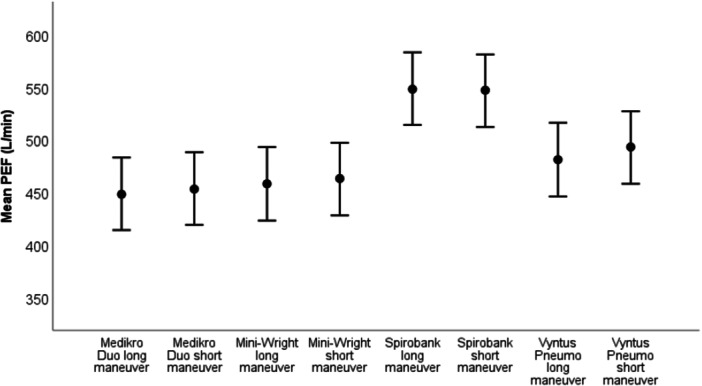
Estimated marginal means of peak expiratory flow (PEF) by device and expiratory maneuver technique derived from the linear mixed‐effects model with device and technique as fixed effects and subject as a random effect. Points represent mean PEF and error bars indicate 95% confidence intervals.

Figure 1 presents the estimated marginal means of PEF by device and expiratory maneuver technique derived from the linear mixed‐effects model with device and technique as fixed effects and subject as a random effect. PEF differed significantly between devices (F(3,133) = 171.8, *p* < 0.001), but not between expiratory maneuver techniques (F(1,133) = 2.24, *p* = 0.137). There was no significant interaction between device and technique (F(3,133) = 0.72, *p* = 0.543). Spirobank yielded the highest PEF while Medikro Duo yielded the lowest. The precise PEF values are provided in Supplement 1.

We also assessed pairwise differences in PEF between devices using estimated marginal means from the linear mixed‐effects model, with results averaged over expiratory maneuver technique. Compared with Mini‐Wright, Spirobank yielded PEF values that were on average 87.1 L/min higher (74.6–99.7, *p* < 0.001), and Vyntus Pneumo 26.5 L/min higher (14.0–39.1, *p* < 0.001), whereas Medikro did not differ significantly from Mini‐Wright (mean difference −9.5 L/min, −22.1–3.1, *p* = 0.269). The pairwise differences between the three spirometers were all statistically significant (all *p* < 0.001). All *p*‐values for pairwise comparisons were Bonferroni‐adjusted.

## 4 DISCUSSION

In this study, expiratory maneuver type (short, explosive *vs.* long, spirometry‐style) did not significantly affect PEF, whereas the measurement device type had a marked effect. All pairwise differences between devices were statistically significant, except for Mini‐Wright and Medikro Duo, which yielded similar PEF values.

The spirometers used in this study differ in several technical aspects that may explain the observed differences in PEF measurements. The laboratory‐based Vyntus Pneumo uses a pneumotachograph, which measures flow based on pressure differences across a resistive element (Jaeger Medical GmbH, 2026). Similarly, Medikro Duo utilizes disposable flow transducers with automated calibration, which are single‐use pneumotachometers (Medikro Oy, 2026). In contrast, Spirobank employs a bidirectional digital turbine flow sensor (Medical International Research, 2026). These differing measurement principles may influence flow measurement, as turbine‐based devices are affected by mechanical inertia (Yeh et al., 1987), while pneumotachographs are sensitive to calibration and environmental conditions, particularly temperature and condensation (Miller & Sigsgaard, 1994). The shared pneumotachograph‐based measurement principle of Vyntus Pneumo and Medikro Duo may partly explain the closer agreement observed between these devices compared with the turbine‐based Spirobank, which represents a fundamentally different measurement approach. Additional factors such as signal processing algorithms and sampling characteristics may also contribute to systematic differences between digital spirometer devices, although detailed information on these aspects is typically proprietary to manufacturers. The Mini‐Wright PEF meter, based on a mechanical spring‐loaded piston system, differs fundamentally from electronic spirometers, as it measures PEF through a purely mechanical mechanism without digital signal processing, which may contribute to differences in measured PEF values (Wright, 1978).

In research settings, PEF measurements obtained from different devices are often used interchangeably. The present findings help to clarify which factors are relevant for achieving measurement equivalence and which are not. As microspirometers have become increasingly prevalent, there has been growing interest in diagnostic home FEV_1_ monitoring as an alternative to traditional home PEF monitoring (Myers et al., 2025). In this context, because our results suggest that differences in expiratory maneuver technique are unlikely to significantly affect absolute PEF values, home spirometry recordings can be used to derive both PEF and FEV_1_ from the same expiratory maneuvers, allowing direct comparison of their diagnostic performance. However, because the largest differences in PEF were observed between the two handheld microspirometers, device selection becomes a critical consideration for such studies. Based on our results, if PEF values comparable to those obtained with a dedicated PEF meter are desired, Medikro Duo may be preferable to Spirobank among the devices evaluated.

Several studies have compared multiple PEF meters and contrasted their results with those from a single spirometer, and most have concluded that PEF values obtained from different devices are not interchangeable (Folgering et al., 1998; Koyama et al., 1998; Takara et al., 2010). In contrast, fewer studies have directly compared PEF derived from different spirometers, and we found no studies comparing the spirometers used in the present study with each other. A study by Bongers & O′Driscoll reported that, using the same expiratory maneuver technique, the Micro Medical Microlab 3000 spirometer yielded significantly higher PEF values than the Vitalograph Spirotach III spirometer, while the Mini‐Wright PEF meter produced the highest values overall (Bongers & O′Driscoll, 2006). In another comparison of two spirometers, Jones and Mullee found that the Vitalograph Escort yielded higher PEF values than the Micro Medical Pocket (Jones & Mullee, 1995). In line with these studies, it is unsurprising that our study also found PEF to vary across devices, since PEF as a parameter is sensitive to resistance of the instrument which is highly variable across devices (Miller et al., 2005).

A limited number of studies have assessed the effect of expiratory maneuver technique on PEF. Bongers & O′Driscoll reported that, overall, a short, explosive maneuver resulted in PEF values that were 8.7% higher than those obtained with a long, spirometry‐style maneuver in adults using various devices (Bongers & O′Driscoll, 2006). Agarwal & Gupta also found a statistically significant difference between the two techniques; however, the absolute difference was only 6 L/min, suggesting limited clinical relevance (Agarwal & Gupta, 2007). Similarly, Wensley et al. reported that in children with and without asthma, spirometry‐style maneuvers yielded PEF values approximately 3% lower, and concluded that this difference was not clinically significant and that PEF derived from spirometry is therefore usable (Wensley et al., 2000). Although the effect of technique did not reach statistical significance in our study, three of the four devices recorded slightly lower PEF values with the long, spirometry‐style maneuver, in line with the aforementioned studies. A small effect may therefore have been present but too small to be detected with the current sample size. However, such a difference would in any case be unlikely to be clinically meaningful.

This study has several strengths. Measurements were highly reliable due to the subjects being experienced in lung function measurements, supervised testing, and rigorous quality control according to ERS criteria for acceptability and repeatability. Furthermore, all devices used in this study are commonly used in clinical practice and meet ERS technical requirements (Graham et al., 2019). By using a linear mixed‐effects model, we accounted for subject‐level variability and the non‐independence of repeated measurements. The primary limitation of this study is the modest sample size. Additional factors that may limit generalizability include the inclusion of only adult subjects, all of whom were healthy, experienced in spirometry, and recruited from a single center. However, the aim of this study was to isolate the effects of expiratory maneuver technique and measurement device. For this specific purpose, the use of a homogeneous, healthy, and experienced study population is a strength rather than a weakness, as it minimizes confounding and allows assessment of a cleaner signal. As an additional limitation, only one PEF meter and three spirometers were evaluated, and therefore the results cannot necessarily be extrapolated to other devices.

In conclusion, PEF measurements obtained using long, spirometry‐style maneuvers appear clinically comparable to those obtained with short, explosive PEF maneuvers, whereas PEF values from different devices are clearly not equivalent. The negligible effect of expiratory maneuver type may be useful in future studies of home‐based spirometry, as it would allow direct comparison of the diagnostic performance of PEF and FEV_1_ derived from the same expiratory maneuvers. However, in such studies, comparison of absolute PEF values between devices must be done with caution.

## CONFLICT OF INTEREST STATEMENT

L.L.C. reports no conflicts of interest. A.T. has received personal fees for lectures from Boehringer Ingelheim, Chiesi, GSK and Orion Pharma. J.K. has received personal fees for advisory board meetings and lectures from Astra Zeneca, Boehringer Ingelheim, Chiesi, GSK, Orion Pharma and Sanofi. L.L. has received personal fees for advisory board meetings and lectures from ALK, Astellas Pharma, Astra Zeneca, Berlin Chemie, Boehringer Ingelheim, Chiesi, GSK, Orion Pharma, and Sanofi.

## Supporting information

Supporting File

## Data Availability

The data that support the findings of this study are available on request from the corresponding author. The data are not publicly available due to privacy or ethical restrictions.
