# Supplementary material for: Effect of device and expiratory maneuver technique on peak expiratory flow measurements
Source: Clin Physiol Funct Imaging. 2026 May 15;46:e70067. doi: 10.1111/cpf.70067 (PMC13177274; doi:10.1111/cpf.70067)
Supplement: Supplementary file 1 — Supporting File [file CPF-46-0-s001.docx]

**Supplement 1**

Table 1 presents the precise PEF values and 95% CIs as estimated marginal means from the linear mixed-effects model, with device and technique included as fixed effects and subject as a random effect.

**Table 1.** Estimated marginal means (95% confidence intervals) of peak expiratory flow (PEF) by device and expiratory maneuver technique derived from the linear mixed-effects model with device and technique as fixed effects and subject as a random effect.

| **Device** | **PEF from short maneuver, L/min (95% CI)** | **PEF from long maneuver, L/min (95% CI)** |
| --- | --- | --- |
| Medikro Duo | 453 (419–488) | 448 (414–483) |
| Spirobank | 547 (512–581) | 548 (514–583) |
| Vyntus Pneumo | 493 (458–527) | 481 (446–516) |
| Mini-Wright | 463 (428–497) | 458 (423–493) |
